# Toward Standardized Monitoring of Patients With Chronic Diseases in Primary Care Using Electronic Medical Records: Systematic Review

**DOI:** 10.2196/10879

**Published:** 2019-05-24

**Authors:** Leandra Falck, Marco Zoller, Thomas Rosemann, Nahara Anani Martínez-González, Corinne Chmiel

**Affiliations:** 1 Institute of Primary Care University of Zurich and University Hospital of Zurich Zurich Switzerland

**Keywords:** monitoring of chronic diseases, indicators, primary care, systematic review, electronic medical record, diabetes mellitus type 2, arterial hypertension, asthma, osteoarthritis, chronic heart failure

## Abstract

**Background:**

Long-term care for patients with chronic diseases poses a huge challenge in primary care. In particular, there is a deficit regarding monitoring and structured follow-up. Appropriate electronic medical records (EMRs) could help improving this but, so far, there are no evidence-based specifications concerning the indicators that should be monitored at regular intervals.

**Objective:**

The aim was to identify and collect a set of evidence-based indicators that could be used for monitoring chronic conditions at regular intervals in primary care using EMRs.

**Methods:**

We searched MEDLINE (Ovid), Embase (Elsevier), the Cochrane Library (Wiley), the reference lists of included studies and relevant reviews, and the content of clinical guidelines. We included primary studies and guidelines reporting about indicators that allow for the assessment of care and help monitor the status and process of disease for five chronic conditions, including type 2 diabetes mellitus, asthma, arterial hypertension, chronic heart failure, and osteoarthritis.

**Results:**

The use of the term “monitoring” in terms of disease management and long-term care for patients with chronic diseases is not widely used in the literature. Nevertheless, we identified a substantial number of disease-specific indicators that can be used for routine monitoring of chronic diseases in primary care by means of EMRs.

**Conclusions:**

To our knowledge, this is the first systematic review summarizing the existing scientific evidence on the standardized long-term monitoring of chronic diseases using EMRs. In a second step, our extensive set of indicators will serve as a generic template for evaluating their usability by means of an adapted Delphi procedure. In a third step, the indicators will be summarized into a user-friendly EMR layout.

## Introduction

In 2016, the World Health Organization estimated that 71% of the overall deaths worldwide occurred due to noncommunicable diseases [1]. The majority of these diseases include cardiovascular diseases, chronic respiratory diseases, and diabetes. In particular, the prevalence of type 2 (non-insulin-dependent) diabetes mellitus, arterial hypertension, asthma, chronic heart failure, and musculoskeletal diseases is increasing rapidly around the world leading to increased multimorbidity and polypharmacy, especially in the older population [1,2]. The burden of these diseases consequently imposes a significant threat to health, quality of life, and economic status in the affected population. Moreover, the regular monitoring of chronic diseases poses huge challenges and requires knowledge and communication skills, as well as the capability of organization and coordination. The chronic care model (CCM) was originally introduced to graphically picture the concept of disease management [3]. The eHealth enhanced chronic care model was subsequently introduced as the means to improve the CCM in view of the progress and development of information and communication technology [4]. This model shows the existing variety of technically well-advanced applications as part of the monitoring process. Too many clinical offices in Switzerland lack basic electronic devices since many general practitioners still use paper-based patient records.

In 2012, 31 European countries were ranked based on the usage of electronic medical records (EMRs) in primary care [5]. In this global ranking of EMR usage, Switzerland ranked number 24. In a Swiss study, only up to 44.8% of the participating primary care physicians reported the usage of EMRs [6]. Therefore, it is currently almost impossible to exchange data with digital applications that are increasingly available and used by patients [6]. To efficiently monitor patients with chronic diseases, a well-structured and organized EMR system is crucial to ensure that all necessary information can be easily entered and retrieved, while no essential information is missed. Surprisingly, there are no evidence-based specifications concerning the indicators that should be monitored at regular intervals. On one hand, there are currently no international standards for the monitoring of patients with chronic diseases by means of EMR in primary care. On the other hand, there are deficits regarding the actual monitoring and structured follow-up. Therefore, we aimed to identify and collect a set of evidence-based indicators that could be used for monitoring patients with chronic conditions at regular intervals in primary care using EMRs.

## Methods

### Systematic Identification and Assessment of Supporting Evidence

We followed the principles of systematic reviews [7] and developed a protocol a priori to guide the identification and assessment of the monitoring indicators.

### Inclusion Criteria

We included clinical guidelines and primary peer-reviewed studies of any design, carried-out mainly in primary care (ie, family health care) patients aged 18 years and older, who were diagnosed with type 2 (non-insulin-dependent) diabetes mellitus, arterial hypertension, asthma, chronic heart failure, or osteoarthritis. The first four diseases are among the most common noninfectious diseases worldwide. Osteoarthritis, in particular, generates a large part of indirect costs [2]. In order to be included, studies must have also reported on indicators that allow the assessment of care and help monitor the status and process of disease for these five chronic conditions. Therefore, we considered disease indicators that help reduce the risk of exacerbation, such as intermediate outcome indicators (eg, hemoglobin A_1c_ [HbA_1c_] for diabetics or blood pressure measurements for hypertensive patients) and process indicators (eg, regular foot care or nutrition counselling). We included studies regardless of whether specific interventions were evaluated. In addition, all studies and clinical guidelines should have been published in English or German.

### Search Methods and Study Identification

We developed a comprehensive search strategy in collaboration with an expert librarian. The librarian conducted the search and produced a set of studies that matched the predefined search criteria. We identified studies published between 2000 and 2015 by applying this strategy in MEDLINE (Ovid), Embase (Elsevier), and the Cochrane Library (Wiley). No restrictions were made regarding the country of origin of the studies. The search strategy included a combination of the concepts and terminology, synonyms and related words for monitoring and for medical, health, electronic, patient, or file records. It also included primary, family, health care, or general practitioner, and the five chronic conditions (ie, type 2 [non-insulin-dependent] diabetes mellitus, arterial hypertension, asthma, chronic heart failure, and osteoarthritis). The focused search also included the terminology indicators, parameter, and management. An example of the full search strategy is available in Multimedia Appendix 1.

We identified additional publications by manually searching the reference lists of included studies and relevant reviews. We also searched for monitoring indicators in the clinical guidelines in order to identify as many indicators as possible and to enable a holistic management of chronic diseases. Given that most guidelines are not indexed in the former medical literature databases, and to identify the clinical guidelines related to any of the five chronic diseases, we searched World Wide Web-based databases, including the National Guideline Clearinghouse for US guidelines [8] and the Arbeitsgemeinschaft der Wissenschaftlichen Medizinischen Fachgesellschaften eV (AWMF) [9] for German guidelines.

### Study Selection and Assessment

For study selection, we created a system to prioritize the studies. One reviewer identified eligible studies by first screening the titles and abstracts of all records retrieved by the searches based on the inclusion criteria. All potentially eligible abstracts were rated manually from one to five stars according to their relevance for this review. The stars were assigned based on whether or not the key terms were mentioned (ie, “indicator,” “monitoring,” “assessment,” “management,” and/or “guideline”). The ranking was assigned as follows:

1. One star: Remote reference to the key terms; no indicators expected in full text.
2. Two stars: Little reference to the key terms; indicators in full text unlikely.
3. Three stars: Reference of at least one key term; indicators in full text possible.
4. Four stars: Reference of at least one key term; indicators in full text very possible.
5. Five stars: Reference of indicators, monitoring, or interval of measuring indicators.

The full text of all studies with an abstract that was rated with at least two stars was obtained, if available, and further evaluated based on the reporting of indicators. For studies where the full text was not available but were deemed important to inform our monitoring tool, we used the data reported in the abstract. When it was necessary, the study team was consulted throughout the evaluation process to confirm the eligibility of indicators.

### Data Extraction and Synthesis

For each included study, we extracted the bibliographic details (ie, author, year, and country of origin), all the monitoring indicators reported, the guideline on which the indicators were based, and the country of origin of the guidelines for each of the five chronic diseases. One reviewer extracted all data, and another reviewer verified the extracted data. We compiled a data profile for each study or guideline, and generated a set of indicators using Microsoft Excel. We report a descriptive summary of the indicators for each of the chronic conditions.

## Results

**Figure 1:**
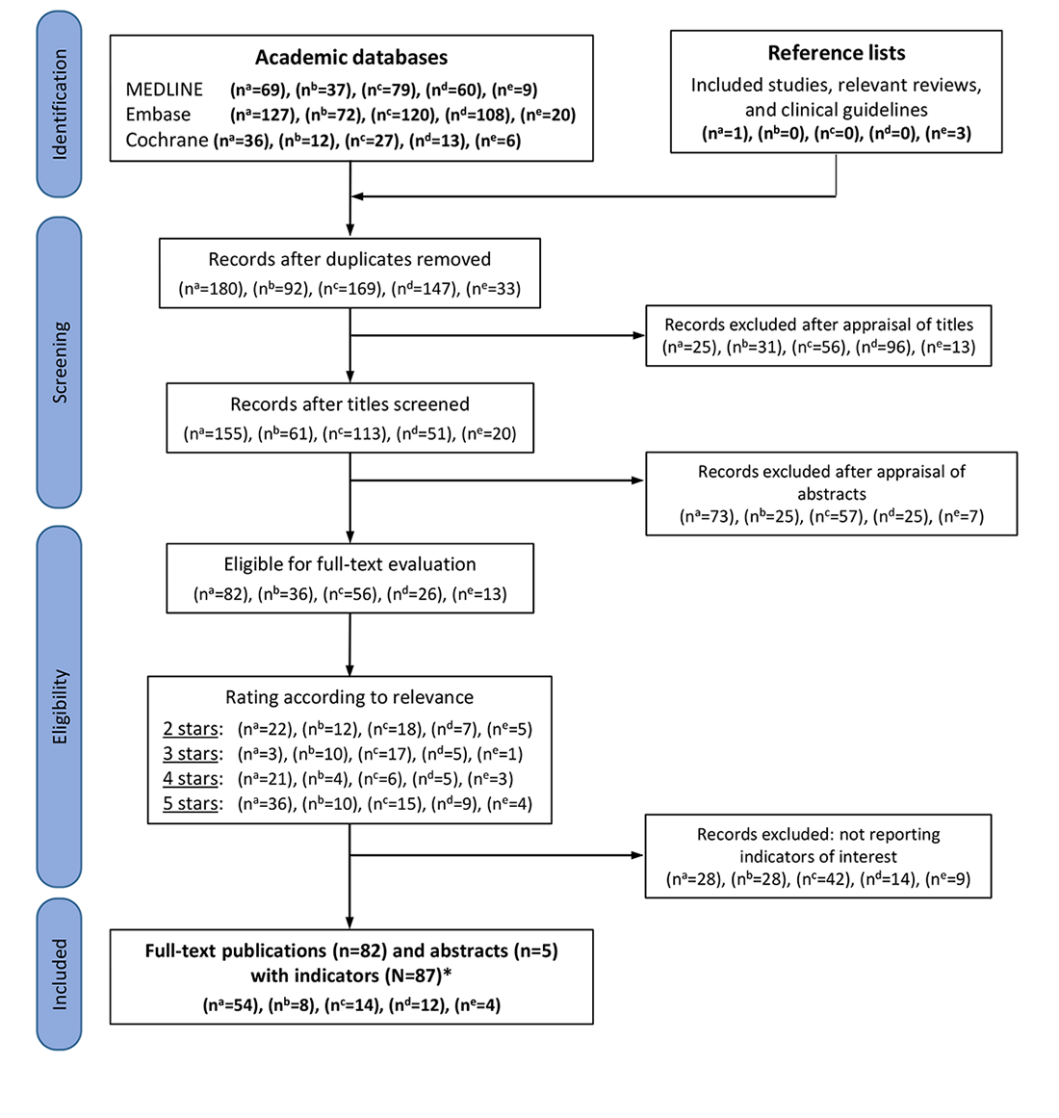
Flowchart demonstrating the identification and selection of evidence. a: type 2 diabetes mellitus; b: asthma; c: arterial hypertension; d: heart failure; e: osteoarthritis; *: 5 of 87 publications (6%) reported indicators for more than one disease of interest.

Our literature searches identified 795 original records (see Figure 1). After deduplication and perusal of titles and abstracts, we screened 621 records (range by disease: 33-180) and excluded 408 records that did not meet our inclusion criteria (eg, focused on specific therapy or medication or did not cover the topic). We examined in detail the full text, where available, of 213 publications (range by disease: 13 to 82).

We included 87 original publications, 5 (6%) in abstract form only, reporting indicators for diabetes mellitus [10-63], asthma [60,64-70], arterial hypertension [10,35,39,71-81], heart failure [33,82-92], and osteoarthritis [93-96]. Multimedia Appendix 2 presents a list of all included studies that reported monitoring indicators for the five chronic conditions. A total of 5 publications (6%) reported indicators for more than one chronic disease [10,33,35,39,60]. The number of included publications by disease with at least one indicator ranged from 4 to 54. Most records (54/87, 62%) were published on type 2 diabetes mellitus, while osteoarthritis was the most underrepresented of the five diseases, with only 4 records (5%). A total of 74 of all 87 included studies (85%) contained process indicators, the most significant type of indicators. Concerning diabetes mellitus, a third of all publications (54/179, 30.2%) reported at least one indicator. For arterial hypertension and heart failure, only 8% (7/87) of all publications reported at least one indicator. Overall, most records used guidelines from the United States, followed by the United Kingdom. For diabetes mellitus, the American Diabetes Association and the National Institute for Health and Care Excellence were the most-used guidelines. The most frequently mentioned indicators for diabetes are presented in Table 1. The indicators for the other four diseases are presented in Multimedia Appendices 3-6.

**Table 1:** Diabetes mellitus indicators that are most frequently mentioned in guidelines and studies. The indicators are sorted first by guidelines and then by studies.

Indicators for diabetes mellitus	Number of guidelines where indicators are mentioned (guidelines)	Number of studies where indicators are mentioned
Fundoscopic examination	7 (a-g)^a^	20 [10,12,13,18,21,23,25,30-32,41,43-46,50-52,60,61]
Height, weight, and body mass index	7 (a-g)	33 [11-16,18,20,21,23-25,27-29,32,33,35,40,41,44,45,48, 49,53-56,58-60,62,63]
Blood pressure measurement	7 (a-g)	45 [11,13,15-27,29-36,39-45,47-49,52,53,55,56,58-63]
10 g monofilament	7 (a-g)	N/A^b^
Hemoglobin A_1c_ (ie, glycated hemoglobin)	7 (a-g)	46 [10,12,13,15-23,26,28-37,39-45,47-54,56-63]
Foot inspection	7 (a-g)	17 [12,15,18,21,23,25,30-32,43-46,50-52,61]
Erectile dysfunction	7 (a-g)	N/A
Albuminuria	7 (a-g)	18 [12,13,18,22,23,25,31,32,35,41,43-46,51,55,61,62]
Lipid profile	7 (a-g)	8 [25,26,30,43,45,46,52,61]
Low-density lipoprotein	N/A	30 [11,12,15,18-20,22-24,29,31-37,41-44,47-49,52-54,63]
High-density lipoprotein	N/A	14 [11,20,23,28,29,33,37,39,49,51,53,54,62,63]
Triglyceride	N/A	15 [20,29,30,33,37,39,48,49,51,53-55,57,62,63]
Creatinine	7 (a-g)	18 [13,15,16,22,25-27,29,33,41,46,51,55,57-60,62]
Alcohol intake	7 (a-g)	2 [24,53]
Neuropathy and history of foot lesion	7 (a-g)	3 [18,20,55]
History of myocardial infarction (ie, cardiovascular disease)	6 (a-f)	2 [18,22]
Foot pulses	6 (a-f)	3 [18,32,60]
Smoking status	6 (a-f)	24 [11-15,18,20,22-24,26,28,29,31,35,41,44,48,50,53, 58-61]
Orthostatic hypotension	5 (a, b, d, e, g)	N/A
Skin inspection	5 (a, b, d, f, g)	N/A
Vibration by 128 Hz tuning fork	5 (a-d, g)	1 [60]
Plasma glucosis	4 (b-d, g)	12 [11,21,24,33,39,45,51,54,55,57,59,63]
Onset of diabetes	3 (b, c, f)	9 [11,18,22,23,28,48,55,58,59]
Indicators appeared in fewer than five guidelines	225	N/A
Indicators appeared in fewer than 10 studies	N/A	76

^a^The letters a-g refer to the guidelines listed in Multimedia Appendices 7-11.

^b^N/A: not applicable.

In total, there were 249 indicators for type 2 diabetes mellitus, 183 for asthma, 335 for arterial hypertension, 231 for chronic heart failure, and 164 for osteoarthritis. The majority of indicators were identified by screening both peer-reviewed articles and clinical guidelines. A few extra indicators were reported only in peer-reviewed articles. That is, clinical guidelines on their own contributed to the great majority of all indicators identified. Surprisingly, only a few guidelines, such as the American guideline for asthma, included a section dedicated to monitoring or follow-up. Most of the guidelines that we screened did not specify the interval at which the indicators should be monitored. Also, in some guidelines, self-monitoring was a big topic for chronic heart disease (ie, weight control), asthma (ie, peak expiratory flow), and type 2 diabetes mellitus (ie, glucose monitoring).

Our systematic review also found that the term “monitoring,” in the sense of long-term patient care, was not widely used. Although publications reported the actual monitoring indicators, the process of monitoring for the different diseases, including, for example, the potential risks associated with overmonitoring, was only scarcely addressed. The publication by Glasziou was the only one giving a broader overview on the topic [97]. Only a handful of publications reported a complete set of indicators that can be used for monitoring, but these were either not specific for primary care or not eligible for implementation in EMRs [98-101].

## Discussion

### Principal Findings

To our knowledge, this study represents the first summary of the existing scientific evidence about the indicators that help standardize the monitoring of chronically ill patients in primary care by the use of EMRs. Long-term care of patients with chronic diseases is challenging and there are deficits regarding their monitoring and structured follow-up. Chronic care often involves collaboration between several people involved in the treatment process. That is only one reason for its complexity. Interpersonal differences in monitoring can decrease the quality of monitoring processes. Surprisingly, there are currently no gold standards or consensus regarding the systematic monitoring of patients with chronic diseases, in particular by means of EMRs. To efficiently monitor patients with chronic diseases, a well-structured and organized EMR system is crucial to ensure that all necessary information can be easily entered and retrieved and that no essential information is missed. Our study is, thus, the first initiative toward the urgent need of standardization for monitoring patients with chronic diseases in primary care.

Our systematic literature review showed that the term “monitoring” in terms of disease management and long-term patient care is not widely used. There is a plethora of literature about quality indicators that might have the potential to improve the outcome of a disease. The Quality and Outcomes Framework (QOF) in the United Kingdom, for example, assesses indicators for such purposes [102]. Beyond identifying indicators that can be easily assessed, such as the indicators used by the QOF, our goal was to summarize the existing literature on all the indicators available for long-term monitoring.

So far, only a few authors have focused on the topic of the monitoring of chronic diseases. According to Glasziou, the process of monitoring aims to establish the response to treatment and to detect both adverse effects and the need to adjust treatment [97]. The process of monitoring can be divided into different phases (ie, pretreatment, during treatment, and after treatment). Each phase requires measurements at different intervals.

When analyzing different diseases, monitoring is probably most widely mentioned in blood pressure management. There are various publications reporting on the optimal way and interval of measuring blood pressure [76,103,104]. However, literature beyond the indicator of blood pressure measurement remains scarce. Regarding diabetes mellitus, there is an extended monitoring tool that was designed as a disease management tool for practice nurses [101]. The tool’s design is based on a traffic light scheme to detect any deficit and need for action. In addition, a detailed guideline on how to monitor the diabetic foot is provided by the International Working Group on the Diabetic Foot [105]. As for bronchial asthma, two study groups have addressed the optimal way and potential problems of finding and evaluating indicators to monitor patients with asthma, including an overview of the most important indicators [98,100]. Similarly, Grypdonck presents a small set of indicators for monitoring patients with osteoarthritis of the knee [93]. Self-monitoring seems to be an important topic concerning osteoarthritis and asthma. An English study conducted by interviewing general practitioners about osteoarthritis showed that the majority of respondents thought monitoring of osteoarthritis is important, even though almost half did not monitor patients at all. Interestingly, more than half of the respondents felt that patients should do self-monitoring [106]. Patient involvement is crucial for monitoring. Particularly, in high-frequency monitoring situations such as chronic heart failure, telecardiological service, including transtelephonic monitoring, reduces the length of hospitalization and improves quality of life [91]. Surprisingly, publications concerning monitoring of chronic heart failure seem to be scarce [90]. The underrepresentation of osteoarthritis and chronic heart failure is also reflected in the number of indicators detected in the primary literature, compared to a large number of records reporting on indicators for type 2 diabetes mellitus. Another topic repeatedly found in the results was the involvement of a clinical practice nurse in monitoring [101,107-109]. The clinical nurse can, for example, fill out a monitoring questionnaire in face-to-face sessions with the patient, on the phone, or even electronically. This could counteract the problem of workload and time constraints as a frequent response to why monitoring is not conducted [106].

### Strengths and Limitations

To our knowledge, this study represents the first scientifically founded recommendation for the standardized long-term monitoring of chronically ill patients in primary care. Usually, systematic reviews only concentrate on primary literature and do not include guidelines in their search strategy, since most guidelines are not indexed in databases. In our study, we explicitly searched for guideline programs such as the National Guideline Clearinghouse for American guidelines and the AWMF for German guidelines. We added a substantial number of manual searches within reference lists and search engines in order to gain a maximal insight of the existing literature. This strategy was worth the extra effort, considering that most relevant indicators were found in guidelines and not in the primary literature. Possible confounders are that publications and guidelines reported in languages other than German and English were excluded.

### Outlook

In a second step, our extensive set of indicators obtained from this work will serve as a generic template for a monitoring tool. By means of an adapted Delphi procedure, the indicators will be further evaluated in terms of their usability. In a third step, the indicators will be summarized into a user-friendly EMR layout.

### Conclusion

This is the first study that systematically summarizes the existing scientific evidence about the standardized long-term monitoring of chronic diseases by means of EMRs. It aims to help improve care for patients with chronic diseases in primary care.

## Appendix

Multimedia Appendix 1 Search strategy for MEDLINE (Ovid), Embase (Elsevier), and the Cochrane Library (Wiley).

Multimedia Appendix 2 List of all included studies, including monitoring indicators for the five chronic conditions.

Multimedia Appendix 3 Asthma indicators mentioned in guidelines and studies.

Multimedia Appendix 4 Arterial hypertension indicators mentioned in guidelines and studies.

Multimedia Appendix 5 Chronic heart failure indicators most frequently mentioned in guidelines and studies.

Multimedia Appendix 6 Osteoarthritis indicators most frequently mentioned in guidelines and studies.

Multimedia Appendix 7 Guidelines screened for indicators for type 2 diabetes mellitus.

Multimedia Appendix 8 Guidelines screened for indicators for asthma.

Multimedia Appendix 9 Guidelines screened for indicators for arterial hypertension.

Multimedia Appendix 10 Guidelines screened for indicators for chronic heart failure.

Multimedia Appendix 11 Guidelines screened for indicators for osteoarthritis.

## Abbreviations

AWMF: Arbeitsgemeinschaft der Wissenschaftlichen Medizinischen Fachgesellschaften eV
CCM: chronic care model
EMR: electronic medical record
HbA_1c_: hemoglobin A_1c_
QOF: Quality and Outcomes Framework
