# Supplementary material for: Toward Standardized Monitoring of Patients With Chronic Diseases in Primary Care Using Electronic Medical Records: Systematic Review
Source: JMIR Med Inform. 2019 May 24;7(2):e10879. doi: 10.2196/10879 (PMC6555125; doi:10.2196/10879)
Supplement: Multimedia Appendix 1 [file medinform_v7i2e10879_app1.docx]

**Appendix 1**

Search strategy for (OVID) MEDLINE*.

| **#** | **Searches** | **Results** |
| --- | --- | --- |
| 1 | (exp Medical Records/ or ((medical or patient) adj3 (record* or file)).ab,ti.) and (general practitioners/ or physicians, family/ or exp Primary Health Care/ or ((general or family or primary) adj3 (doctor* or physician* or practi*)).ab,ti.) and (exp Automatic Data Processing/ or indicator.ab,ti. or managment.ab,ti. or monitoring.ab,ti. or parameter.ab,ti. or ((clinical or laboratory) adj3 (data or assess* or finding* or observ* or monitor* or examin* or check* or control*)).ab,ti. or ((data or information) adj6 (set or extract* or analys* or retriev* or yield* or gather*)).ab,ti. or (data adj6 (standard* or normali* or form*)).ab,ti. or ("data from" adj6 (file or record)).ab,ti.) | 2088 |
| 2 | limit 1 to (yr="2000 -Current" and (english or german)) | 1593 |
| 3 | exp Diabetes Mellitus, Type 2/ | 91561 |
| 4 | 2 and 3 | 74 |
| 5 | (self monitoring or (monitoring adj3 glucose)).ab,ti. | 9074 |
| 6 | 4 not 5 | 69 |
| 7 | exp Hypertension/ | 215840 |
| 8 | 2 and 7 | 79 |
| 9 | exp Heart Failure/ | 91040 |
| 10 | 2 and 9 | 22 |
| 11 | exp heart failure/ or exp myocardial ischemia/ | 442513 |
| 12 | 2 and 11 | 60 |
| 13 | 12 not 10 | 38 |
| 14 | exp Asthma/ | 108981 |
| 15 | 2 and 14 | 37 |
| 16 | exp Arthritis/ | 209132 |
| 17 | 2 and 16 | 16 |

*Similar search strategies were applied in Embase and Cochrane.
