# Supplementary material for: Toward Standardized Monitoring of Patients With Chronic Diseases in Primary Care Using Electronic Medical Records: Systematic Review
Source: JMIR Med Inform. 2019 May 24;7(2):e10879. doi: 10.2196/10879 (PMC6555125; doi:10.2196/10879)
Supplement: Multimedia Appendix 2 [file medinform_v7i2e10879_app2.docx]

**Appendix 2**

**List of all included studies including monitoring indicators for the five chronic conditions.**

| **First author** | **Year of publication** | **Country of origin** | | **Guideline used in the publication** | **Country of origin of guideline** | **Reference number** |
| --- | --- | --- | --- | --- | --- | --- |
| **Diabetes mellitus** | | | | | | |
| Suija | 2015 | Estonia | | Organisation for Economic Co-operation and Development (OECD), American Diabetes Association (ADA), World Health Organization WHO, European Society of Cardiology (ESC), National Institute for Health and Care Excellence (NICE) | various | [10] |
| Shah | 2015 | United Kingdom | | unknown |  | [11] |
| Devkota | 2015 | USA | | American Diabetes Association (ADA) | USA | [12] |
| Barkhuysen | 2014 | Netherlands | | Dutch College of General Practitioners | Netherlands | [13] |
| Szczech | 2014 | USA | | Kidney Disease Outcomes Quality Initiative (KDOQI) | USA | [14] |
| van Melle | 2014 | Netherlands | | Dutch College of General Practitioners | Netherlands | [15] |
| Djalali | 2014 | Switzerland | | Quality and Outcomes Framework indicator (QOF) | United Kingdom | [16] |
| Goff | 2014 | USA | | unknown |  | [17] |
| Vidal-Pardo | 2013 | Spain | | "Plan de Saude" (Health plan of Galicia) | Spain | [18] |
| Sidorenkov | 2013 | Netherlands | | Quality and Outcomes Framework indicator (QOF), Dutch College of General Practitioners | United Kingdom, Netherlands | [19] |
| Winkley | 2013 | United Kingdom | | unknown |  | [20] |
| Gavran | 2012 | Bosnia/Herzegovina | | The Committee for Practice Guidelines (CPG) | Europe | [21] |
| Knudsen | 2012 | Denmark | | Danish National guidelines | Denmark | [22] |
| Mata-Cases | 2012 | Spain | | RedGDPS (Spain) | Spain | [23] |
| Nouwens | 2012 | Netherlands | | Dutch College of Family Physicians | Netherlands | [24] |
| Satman | 2012 | Turkey | | Standard Diabetes Management Procedures of Turkey | Turkey | [25] |
| Marley | 2012 | Australia | | unknown |  | [26] |
| Patapas | 2012 | Canada | | unknown |  | [27] |
| Staff | 2012 | Asutralia | | United Kingdom Prospective Diabetes Study | United Kingdom | [28] |
| Hill | 2012 | Ireland | | indicators selected by authors |  | [29] |
| Alfadda | 2011 | Saudi Arabia | | American Diabetes Association (ADA) | USA | [30] |
| Dickerson | 2011 | USA | | Diabetes Recognition Program (DRP) by the National Committee for Quality Assurance (NCQA) | USA | [31] |
| Vidal Pardo | 2011 | Spain | | "Plan de Saude" (Health plan of Galicia) | Spain | [32] |
| Weenink | 2011 | Netherlands | | unknown |  | [33] |
| Gladstone | 2011 | USA | | Canadian Practice Guidelines | Canada | [34] |
| Holbrook | 2011 | Canada | | unspecific cardiovascular risk factors |  | [35] |
| O'Connor | 2011 | USA | | unknown |  | [36] |
| Sundquist | 2011 | SWE | | Swedish National Guidelines | Sweden | [37] |
| Reddy | 2010 | Australia | | Quality and Outcomes Framework indicator (QOF) | United Kingdom | [38] |
| Samoutis | 2010 | Cyprus | | American Diabetes Association (ADA), St. Vincent Declaration | USA, Europe | [39] |
| Shah | 2010 | Canada | | Canadian Diabetes Association | Canada | [40] |
| Petrazzuoli | 2010 | Italy | | Quality and Outcomes Framework indicator (QOF) | United Kingdom | [41] |
| Sperl-Hillen | 2010 | USA | | American Diabetes Association (ADA) | USA | [42] |
| Pedersen | 2009 | Greenland | | Danish National Indicator Project | Denmark | [43] |
| Holbrook | 2009 | Canada | | Canadian Diabetes Association and American Diabetes Association (ADA) | Canada, USA | [44] |
| Moharram | 2008 | Saudi Arabia | | Canadian Diabetes Association | Canada | [45] |
| Novo | 2008 | Bosnia/Herzegovina | | Canadian Diabetes Association | Canada | [46] |
| Samuels | 2008 | USA | | American Diabetes Association (ADA) | USA | [47] |
| Smith | 2008 | USA | | American Diabetes Association (ADA), National Committee for quality assurance | USA | [48] |
| Voorham | 2008 | NL | | National Guidelines of Netherlands | Netherlands | [49] |
| Wens | 2007 | Belgium | | various guidelines | Belgium, Netherlands, Germany, United Kingdom, France | [50] |
| Nitiyanant | 2007 | Thailand | | unknown |  | [51] |
| Herrin | 2006 | USA | | National Diabetes Quality Improvement Alliance guidelines | USA | [52] |
| Wan | 2006 | Australia | | Australian guidelines for diabetes management in general practice, National Institute for Health and Care Excellence (NICE) | Australia, United Kingdom | [53] |
| Al Khaja | 2005 | Bahrain | | The Seventh Report of the Joint National Committee (JNC-7) | USA | [54] |
| Cueto-Manzano | 2005 | Mexico | | unknown |  | [55] |
| Lusignan | 2005 | United Kingdom | | National Institute for Health and Care Excellence (NICE) | United Kingdom | [56] |
| Sequeira | 2004 | Bahrain | | The Sixth Report of the Joint National Committee (JNC-6)/International Society of Hypertension (ISH) | USA, international | [57] |
| Wermeille | 2004 | Switzerland | | unknown |  | [58] |
| Goudswaard | 2003 | Netherlands | | Dutch College of General Practitioners | Netherlands | [59] |
| Campbell | 2002 | United Kingdom | | Developed within study |  | [60] |
| Parchman | 2002 | USA | | American Diabetes Association (ADA) | USA | [61] |
| Renders | 2001 | Netherlands | | Dutch College of General Practitioners | Denmark | [62] |
| Linmans | 2001 | Netherlands | | unknown |  | [63] |
| **Asthma** | | | | | | |
| Minard | 2014 | | Canada | Asthma Care Map | Canada | [64] |
| Lim | 2012 | | USA | National Assessment of Educational Progress (NAEP) | USA | [65] |
| Lougheed | 2012 | | Canada | Canadian Asthma Consensus Guidelines/Canadian Thoracic Society | Canada | [66] |
| Oei | 2011 | | Australia | unknown |  | [67] |
| Nokela | 2010 | | Sweden | unknown |  | [68] |
| Yawn | 2008 | | USA | APGAR tool/ National Heart, Lung, and Blood Institute (NHLBI) | USA | [69] |
| Baddar | 2006 | | Oman | Oman’s national Manual for the Management of Asthma in Adults | Oman | [70] |
| Campbell | 2002 | | United Kingdom | indicators selected by authors |  | [60] |
| **Arterial hypertension** | | | | | | |
| Suija | 2015 | | Estonia | Organisation for Economic Co-operation and Development (OECD), European Society of Cardiology (ESC), World Health Organization (WHO)/ International Society of Hypertension (ISH) | international, Europe | [10] |
| Hasselstrom | 2014 | | Sweden | unknown |  | [71] |
| Tong | 2012 | | Malaysia | Clinical Practice Guideline for Hypertension in Malaysia | Malaysia | [72] |
| Holbrook | 2011 | | Canada | unknown |  | [35] |
| Samoutis | 2010 | | Cyprus | Report of the Joint National Committee (JNC), the European guidelines on cardiovascular disease prevention and the European Society of Hypertension, European Society of Cardiology Guidelines. | USA, Europe | [39] |
| Pavlik | 2009 | | USA | The Seventh Report of the Joint National Committee (JNC-7) | USA | [73] |
| Chan | 2006 | | China | Eli Lilly National clinic audit center, Report of the Joint National Committee (JNC) | United Kingdom, USA | [74] |
| Asnani | 2005 | | Jamaica | Ministry of Health | Jamaica | [75] |
| Rabinowitz | 2005 | | Israel | American Heart Association (AHA)/American College of Cardiology (ACC), National Cholesterol Education Program | USA | [76] |
| Mitchell | 2005 | | United Kingdom | unknown |  | [77] |
| Alli | 2005 | | Italy | unknown |  | [78] |
| Tierney | 2004 | | USA | unknown |  | [79] |
| Lackland | 2004 | | USA | The Seventh Report of the Joint National Committee (JNC-7) | USA | [80] |
| Frijling | 2003 | | Netherlands | Dutch college of General Practitioners | Netherlands | [81] |
| **Heart failure** | | | | | | |
| Amarasingham | 2013 | | USA | unknown |  | [82] |
| Logeart | 2013 | | France | European Society of Cardiology (ESC) | Europe | [83] |
| Weenink | 2011 | | Netherlands | unknown |  | [33] |
| Lind | 2011 | | Sweden | unknown |  | [84] |
| Korb | 2010 | | Germany | Deutsche Gesellschaft für Allgemeinmedizin und Familienmedizin (DEGAM)-Leitlinie | Germany | [86] |
| Maddocks | 2010 | | Canada | Heart failure management incentive (ministry of health and long-term care) | Canada | [85] |
| Fonarow | 2010 | | USA | American College of Cardiology/American Heart Association | USA | [87] |
| Vercauteren | 2009 | | Belgium | unknown |  | [88] |
| Majeed | 2005 | | United kingdom | Quality and Outcomes Framework indicator (QOF) | United Kingdom | [89] |
| Roth | 2004 | | Israel | unknown |  | [91] |
| Subramanian | 2004 | | USA | Kansas City Cardiomyopathy Questionnaire | USA | [90] |
| Gnani | 2004 | | United Kingdom | Quality and Outcomes Framework indicator (QOF) | United Kingdom | [92] |
| **Osteoarthritis** | | | | | | |
| Grypdonck | 2014 | | Belgium | indicators selected by authors |  | [93] |
| Jansen | 2010 | | Netherlands | Osteoarthritis of the hip and knee for physical therapist | Netherlands | [94] |
| MacLean | 2004 | | USA | indicators selected by authors |  | [95] |
| Peat | 2002 | | United Kingdom | indicators selected by authors |  | [96] |
