# Supplementary material for: Toward Standardized Monitoring of Patients With Chronic Diseases in Primary Care Using Electronic Medical Records: Systematic Review
Source: JMIR Med Inform. 2019 May 24;7(2):e10879. doi: 10.2196/10879 (PMC6555125; doi:10.2196/10879)
Supplement: Multimedia Appendix 3 [file medinform_v7i2e10879_app3.docx]

**Appendix 3**

Asthma indicators mentioned in guidelines and studies. The indicators are sorted first by guidelines and then by studies.

| **indicators for asthma** | **appeared in guidelines** | **appeared in studies** |
| --- | --- | --- |
| Fev_1_/FVC Ratio | **6** (a-f) | - |
| lung function | **6** (a-f) | **3** [64, 66, 69] |
| Daytime asthma symptoms | **6** (a-f) | **4** [60, 64, 66, 69] |
| Any night waking due to asthma | **5** (a, c-f) | **5** [60, 64, 66, 68, 69] |
| Any activity limitation due to asthma | **5** (a, c-f) | **4** [60, 64, 66, 69] |
| FEV_1_ | **5** (a-c, e, f) | **1** [68] |
| PEF (self-monitoring) | **5** (a-c, e, f | **4** [60, 64, 68, 70] |
| wheezing | **4** (b-e) | **2** [64, 69] |
| cough | **4** (b-e) | **2** [64, 69] |
| Reliever needed for symptoms more than twice per week | **4** (a, c, d, f) | **2** [64, 68] |
| smoking habit | **4** (a-d) | **3** [60, 65, 68] |
| auscultation | **4** (a, b, d, e) | - |
| bronchial provocation test | **4** (a, b, d, e) | - |
| variation in lung function | **4** (a, d-f) | - |
| check inhaler technique | **3** (a-c) | **4** [60, 64, 69, 70] |
| trigger of symptoms | **3** (a, c, d) | **3** [64, 69, 70] |
| hospital stays or emergency department visits since last visit | **1** (c) | **3** [64, 66, 70] |
| medication history | **2** (a,c) | **3** [64, 69, 70] |
| smoking cessation advice | - | **3** [60, 64, 66] |
| check adherence | **2** (a,c) | **3** [64, 68, 70] |
| asthma action plan | - | **3** [64, 66, 69] |
| indicators appeared in less than 4 guidelines | **162** |  |
| indicators appeared in less than 3 studies |  | **38** |

Letters a-f refers to the guidelines listed in Appendix 8; FEV_1_/FVC Ratio: Tiffeneau-Pinelli index. FEV_1_: Forced expiratory volume at one second. PEF: peak expiratory flow.
