# Supplementary material for: Toward Standardized Monitoring of Patients With Chronic Diseases in Primary Care Using Electronic Medical Records: Systematic Review
Source: JMIR Med Inform. 2019 May 24;7(2):e10879. doi: 10.2196/10879 (PMC6555125; doi:10.2196/10879)
Supplement: Multimedia Appendix 4 [file medinform_v7i2e10879_app4.docx]

**Appendix 4**

Arterial hypertension indicators mentioned in guidelines and studies. The indicators are sorted first by guidelines and then by studies.

| **indicators for arterial hypertension** | **appeared in guidelines** | **appeared in studies** |
| --- | --- | --- |
| smoking habit | **8** (a-h) | **11** [35, 71-79, 81] |
| blood pressure | **8** (a-h) | **9** [10, 35, 39, 71-73, 75, 77-79] |
| choose correct cuff | **8** (a-h) | **1** [75] |
| standing blood pressure | **8** (a-h) | - |
| electrocardiogramm | **8** (a-h) | **2** [72, 75] |
| fasting blood glucose and or HbA1c | **8** (a-h) | **6** [39, 71, 72, 75, 79, 80] |
| serum totalcholesterine | **8** (a-h) | **8** [10, 39, 71, 72, 74, 75, 79, 80] |
| high density lipoprotein | **8** (a-h) | **5** [39, 71, 72, 76, 80] |
| creatinine | **8** (a-h) | **4** [71, 75, 79, 80] |
| potassium | **8** (a-h) | **3** [75, 79, 80] |
| age | **7** (a-f, h) | **2** [76, 78] |
| history of chronic kidney disease | **7** (a, c-h) | **2** [78, 79] |
| recreational drug use | **7** (a, c-h) | - |
| current medication | **7** (a, c-h) | **3** [73, 75-77] |
| family history | **7** (a-d, g, h) | **3** [72, 74, 76] |
| family history for coronary heart disease | **7** (a-d, g, h) | **3** [72, 74, 76] |
| BMI/weight | **7** (a-f, h) | **8** [35, 71, 72, 74-76, 78, 79] |
| resting comfortably for several minutes prior to blood pressure measurement | **7** (a, b, d-h) | - |
| measure blood pressure on both arms | **7** (a-e, g,h) | - |
| Home blood pressure monitoring or ambulatory blood pressure monitoring if suspected white coat hypertension | **7** (a-g) | - |
| eye examination | **7** (a-d, e-h) | 3 [72, 74, 75] |
| echocardiography if needed | **7** (a-c, e-h) | - |
| low density lipoprotein | **7** (a, b, d-h) | **6** [35, 39, 71, 72, 76, 80] |
| triglycerides | **7** (a, b, d-h) | **6** [39, 71, 72, 75, 76, 80] |
| Assessment of microalbuminuria | **7** (a, b, d-h) | **4** [71, 72, 79, 80] |
| sedentary lifestyle | **6** (a, d-h) | **5** [35, 74-76, 78] |
| diabetes mellitus in history | **6** (a, c, d, f-h) | **6** [72, 74, 76-79] |
| alcohol | **3** (e, g, h) | **4** [74, 75, 78, 79] |
| history of coronary heart disease/cardiovascular disease/stroke | **5** (a, d-g) | **2** [77-79] |
| indicators appeared in less than 7 guidelines | **307** |  |
| indicators appeared in less than 3 studies |  | **58** |

Letters a-h refer to the guidelines listed in Appendix 9; BMI: body mass index.
