# Supplementary material for: Toward Standardized Monitoring of Patients With Chronic Diseases in Primary Care Using Electronic Medical Records: Systematic Review
Source: JMIR Med Inform. 2019 May 24;7(2):e10879. doi: 10.2196/10879 (PMC6555125; doi:10.2196/10879)
Supplement: Multimedia Appendix 5 [file medinform_v7i2e10879_app5.docx]

**Appendix 5**

Chronic heart failure indicators most frequently mentioned in guidelines and studies. The indicators are sorted first by guidelines and then by studies.

| **indicators for chronic heart failure** | **appeared in guidelines** | **appeared in studies** |
| --- | --- | --- |
| sodium | **6** (a-f) | **4** [33, 85, 87, 89] |
| potassium | **6** (a-f) | **3** [33, 85, 89] |
| creatinine | **6** (a-f) | **5** [33, 82, 83, 85, 87] |
| daily weight measurement | **5** (a, b, d-f) | **3** [84, 88, 91] |
| changes in weight | **5** (a, b, d-f) |  |
| depression | **5** (a, c-f) | - |
| address palliative or hospice care | **5** (a, c, d-e) | - |
| NYHA-classification | **5** (a, c-f) | **2** [87, 90] |
| pulse rate | **5** (a-c, e-f) | **3** [83, 85, 87] |
| exercise capacity | **5** (a, b, d-f) | **2** [86, 90] |
| erectile dysfuntion/sexual activity | **4** (a, d, e, f) | - |
| measure blood pressure | **4** (a, b, d, e) | **5** [33, 85, 87, 89, 92] |
| measure daily blood pressure |  | **3** [84, 88, 91] |
| electrocardiography | **4** (a, d-f) | **4** [85, 86, 89, 92] |
| echocardiogram with doppler | **4** (a, d-f) | **6** [85-89, 92] |
| NT-proBNP | **3** (d-f) | **5** [82, 83, 86-88] |
| chest radiography if necessary | **3** (d-f) | **4** [85, 86, 89, 92] |
| BMI | **2** (e, f) | **3** [33, 85, 89] |
| history/signs of diabetes mellitus | **3** (a, d, f) | **3** [82, 87, 89] |
| number of indicators that appeared in less than 4 guidelines | **213** |  |
| number of indicators that appeared in less than 3 studies |  | **47** |

Letters a-f refer to the guidelines listed in Appendix 10; NT-proBNP: N-terminal prohormone Brain natriuretic peptide. BMI: body mass index.
