# Supplementary material for: Toward Standardized Monitoring of Patients With Chronic Diseases in Primary Care Using Electronic Medical Records: Systematic Review
Source: JMIR Med Inform. 2019 May 24;7(2):e10879. doi: 10.2196/10879 (PMC6555125; doi:10.2196/10879)
Supplement: Multimedia Appendix 6 [file medinform_v7i2e10879_app6.docx]

**Appendix 6**

Osteoarthritis indicators most frequently mentioned in guidelines and studies. The indicators are sorted first by guidelines and then by studies.

| **indicators for osteoarthritis** | **appeared in guidelines** | **appeared in studies** |
| --- | --- | --- |
| tenderness | **4** (a-d) |  |
| significant loss of range of movement | **4** (a-d) |  |
| assessment of functional status |  | **2** [94, 95] |
| mood | **3** (a-c) |  |
| health believes | **3** (a-c) |  |
| expectations, concerns, ideas | **3** (a-c) |  |
| stiffness | **3** (a, c, d) |  |
| stress pain | **3** (a, c, d) |  |
| activity of daily living | **3** (a, b, d) |  |
| Hobbies | **3** (a-c) |  |
| history of trauma | **3** (a, c, d) |  |
| swelling | **3** (a, c, d) |  |
| redness | **3** (a-c) |  |
| deformity | **3** (a, b, d) |  |
| advise on losing and maintaining weight |  | **2** [93, 95] |
| assessment of pain |  | **2** [94, 95] |
| number of indicators that appeared in less than 3 guidelines | **148** |  |
| number of indicators that appeared in only one study |  | **38** |

Letters a-f refer to the guidelines listed in Appendix 11.
