# Supplementary material for: Toward Standardized Monitoring of Patients With Chronic Diseases in Primary Care Using Electronic Medical Records: Systematic Review
Source: JMIR Med Inform. 2019 May 24;7(2):e10879. doi: 10.2196/10879 (PMC6555125; doi:10.2196/10879)
Supplement: Multimedia Appendix 7 [file medinform_v7i2e10879_app7.docx]

**Appendix 7**

Guidelines screened for indicators for diabetes mellitus type2.

| **Diabetes mellitus** | **Year (last update)** | **editor/publisher** | **country** |  |
| --- | --- | --- | --- | --- |
| Standards of Medical Care in Diabetes | 2014 | American Diabetes Association (ADA) | USA | a |
| Nationale VersorgungsLeitlinien | 2013, 2015 | Bundesärztekammer (BÄK), Kassenärztliche Bundesvereinigung (KBV), Arbeitsgemeinschaft der Wissenschaftlichen Medizinischen Fachgesellschaften (AWMF)  (AWMF Institute for Medical Knowledge Management) | Germany | b |
| National Institute for Health and Care Excellence (NICE) | 2014 | Royal College of Physicians | England | c |
| Global Guideline for Type 2 Diabetes | 2012 | International Diabetes Federation | International | d |
| General practice management of type 2 diabetes | 2014 | The Royal Australian College of General Practitioners | Australa | e |
| Clinical Practice Guidelines | 2013 | Canadian Diabetes Association | Canada | f |
| MediX-Guideline zu Diabetes mellitus | 2013 | MediX | Schweiz | g |
