# Supplementary material for: Toward Standardized Monitoring of Patients With Chronic Diseases in Primary Care Using Electronic Medical Records: Systematic Review
Source: JMIR Med Inform. 2019 May 24;7(2):e10879. doi: 10.2196/10879 (PMC6555125; doi:10.2196/10879)
Supplement: Multimedia Appendix 8 [file medinform_v7i2e10879_app8.docx]

**Appendix 8**

Guidelines screened for indicators for asthma.

| **Asthma** | **Year (last update)** | **editor/publisher** | **country** |  |
| --- | --- | --- | --- | --- |
| Global strategy for Asthma Management and Prevention | 2015 | Global Initiative for Asthma (GINA) | international | a |
| Asthma: diagnosis and monitoring of asthma in adults, children and young people | Draft for Consultation 2015 | National Institute for Health and Care Excellence (NICE) | England | b |
| Guidelines for the Diagnosis and Management of Asthma | 2007 | National Heart, Lung,  and Blood Institute (NHBLI) | USA | c |
| Australian Asthma Handbook  (Quick Reference Guide) | 2014 | The Royal Australian College of General Practitioners | Australia | d |
| Leitlinien zu Diagnostik und Therapie von Patienten mit Asthma | 2006 | Deutsche Atemwegsliga, Deutsche Gesellschaft für Pneumologie und Beatmungsmedizin e.V. | Germany | e |
| MediX-Guideline zu Asthma bronchiale | 2011 | MediX | Switzerland | f |
