# Supplementary material for: Toward Standardized Monitoring of Patients With Chronic Diseases in Primary Care Using Electronic Medical Records: Systematic Review
Source: JMIR Med Inform. 2019 May 24;7(2):e10879. doi: 10.2196/10879 (PMC6555125; doi:10.2196/10879)
Supplement: Multimedia Appendix 9 [file medinform_v7i2e10879_app9.docx]

**Appendix 9**

Guidelines screened for indicators for arterial hypertension.

| **Arterial hypertension** | **Year (last update)** | **editor/publisher** | **country** |  |
| --- | --- | --- | --- | --- |
| Leitlinien für das Management der arteriellen Hypertonie  (Guidelines for management of arterial hypertension) | 2013 | Deutsche Gesellschaft für Kardiologie-, Herz- und Kreislaufforschung (DGK) (German Cardiac Society) and Deutsche Hochdruckliga e.V. DHL® Deutsche Gesellschaft für Hypertonie und Prävention | Germany | a |
| Practice Guidelines for the Management of Arterial Hypertension | 2007 | The European Society of Cardiology (ESC) and European Society of Hypertension (ESH) | Europe | b |
| Clinical management of primary hypertension in adults | 2013 | National Institute for Health and Clinical Excellence (NICE) | England | c |
| The Seventh Report of the Joint National Committee on Prevention, Detection, Evaluation, and Treatment of High Blood Pressure | 2003 | National Heart, Lung, and Blood Institute (NHLBI) | USA | d |
| The 2015 Canadian Hypertension Education Program Recommendations | 2015 | Hypertension Canada | Canada | e |
| Clinical Practice Guidelines for the Management of Hypertension in the Community | 2014 | The American Society of Hypertension and the International Society of Hypertension (ISH) | USA, international | f |
| Guide to management of hypertension 2008 (Assessing and managing raised blood pressure in adults) | 2010 | National Heart Foundation of Australia | Australia | g |
| Arterielle Hypertonie Empfehlungen für Ärzte | 2015 | Swiss Society of Hypertension (Schweizerische Hypertonie Gesellschaft) | Switzerland | h |
