# Supplementary material for: Toward Standardized Monitoring of Patients With Chronic Diseases in Primary Care Using Electronic Medical Records: Systematic Review
Source: JMIR Med Inform. 2019 May 24;7(2):e10879. doi: 10.2196/10879 (PMC6555125; doi:10.2196/10879)
Supplement: Multimedia Appendix 10 [file medinform_v7i2e10879_app10.docx]

**Appendix 10**

Guidelines screened for indicators for chronic heart failure.

| **Chronic heart failure** | **Year (last update)** | **editor/publisher** | **country** |  |
| --- | --- | --- | --- | --- |
| Nationale VersorgungsLeitlinie | 2011 | Bundesärztekammer (BÄK), Kassenärztliche Bundesvereinigung (KBV), Arbeitsgemeinschaft der Wissenschaftlichen Medizinischen Fachgesellschaften (AWMF)  (AWMF Institute for Medical Knowledge Management) | Germany | a |
| Anforderungen an ein Modul „Chronische Herzinsuffizienz“ für strukturierte Behandlungsprogramme für Koronare Herzkrankheit (KHK) | -- | Empfehlungen des Gemeinsamen Bundesausschusses gemäss §137f Abs. 2 SGB V für die Rechtsverordnung nach § 266 Abs. 7 SGB V | Germany | b |
| Chronic Heart Failure; National clinical guideline for diagnosis and management in primary and secondary care | 2010 | National Institute for Health and Clinical Excellence (NICE) | England | c |
| ESC Guidelines for the diagnosis and treatment of acute and chronic heart failure 2012 | 2012 | The European Society of Cardiology (ESC) | Europe | d |
| Guideline for the Management of Heart Failure | 2013 | The American College of Cardiology Foundation and the American Heart Association | USA | e |
| Guidelines for the prevention, detection and management of chronic heart failure in Australia | 2011 | National Heart Foundation of Australia | Australia | f |
