# Supplementary material for: Toward Standardized Monitoring of Patients With Chronic Diseases in Primary Care Using Electronic Medical Records: Systematic Review
Source: JMIR Med Inform. 2019 May 24;7(2):e10879. doi: 10.2196/10879 (PMC6555125; doi:10.2196/10879)
Supplement: Multimedia Appendix 11 [file medinform_v7i2e10879_app11.docx]

**Appendix 11**

Guidelines screened for indicators for osteoarthritis.

| **Osteoarthritis** | **Year (last update)** | **editor/publisher** | **country** |  |
| --- | --- | --- | --- | --- |
| Osteoarthritis; Care and management in adults | 2014 | National Institute for Health and Clinical Excellence (NICE) | England | a |
| EULAR recommendations for the non-pharmacological core management of hip and knee osteoarthritis | 2013 | The European League against Rheumatism | Europe | b |
| Guideline for the non-surgical management of hip and knee osteoarthritis | 2009 | The Royal Australian College of General Practitioners | Australia | c |
| MediX-Guideline zu Arthrose | 2013 | MediX | Schweiz | d |
